# Age-Related Patterns in Child-to-Parent Violence Across Adolescence and Emerging Adulthood

**DOI:** 10.3390/ejihpe16050070

**Published:** 2026-05-17

**Authors:** María J. Navas-Martínez, Lourdes Contreras, Nazaret Bautista-Aranda, M. Carmen Cano-Lozano

**Affiliations:** 1 Department of Psychology, University of Jaén, 23071 Jaén, Spain; lmcontre@ujaen.es (L.C.); mccano@ujaen.es (M.C.C.-L.); 2 Department of Developmental and Educational Psychology, Jaume I University, 12006 Castellón, Spain; nbautist@uji.es

**Keywords:** child-to-parent violence, patterns, age, sex, adolescents, young adults

## Abstract

Background: The aim of this study was to examine the pattern of child-to-parent violence (CPV) across a broad age range, from early adolescence to late emerging adulthood. Specifically, the objectives were to analyze the linear and quadratic relationships between CPV types (psychological, physical, financial, and control/domain behaviors) and age, as well as to examine the interaction of sex within this relationship. Methods: A total of 1959 adolescents (13–17 years) and 1046 young adults (18–25 years) completed, respectively, the adolescent and young adult versions of the Child-to-Parent Violence Questionnaire (CPV-Q). Results: Age was curvilinearly associated with psychological CPV (increasing until approximately age 19 and then decreasing), positively linearly associated with financial CPV (increasing with age), and negatively linearly associated with control/domain behaviors (decreasing with age). No significant association was found between age and physical CPV. Furthermore, boys and girls showed different age-related patterns in some CPV types. Conclusions: These findings suggest that CPV does not disappear after adolescence, and that the pattern is not uniform throughout development nor the same for boys and girls. The results (1) underscore the importance of studying CPV considering developmental stage, sex, and the specific CPV types, and (2) may contribute to facilitate the early detection of CPV, anticipating changes in violence patterns, and guiding prevention strategies tailored to each developmental stage.

## 1. Introduction

Violence by children (sons and daughters) toward their parents, or child-to-parent violence (hereinafter CPV), is currently regarded as a serious social problem with legal consequences and significant impacts on the deterioration of the health and psychosocial well-being of affected families (Bautista-Aranda et al., 2025; Beckmann et al., 2021; Cano-Lozano et al., 2024; Simmons et al., 2020). This form of violence is defined as any conscious, intentional, and repeated act (Pereira et al., 2017) of physical, psychological, and/or financial violence (Cottrell, 2001) aimed at gaining power, control (Cottrell, 2001) and domain (Howard & Rottem, 2008) over parents or parental figures (Molla-Esparza & Aroca-Montolío, 2018).

The purpose of this study is to analyze age-related patterns of CPV across adolescence and emerging adulthood. CPV constitutes a specific form of family violence characterized by the upward direction of violent behavior (from children to parents), which distinguishes it from other forms of family violence typically perpetrated by adults. Given that CPV occurs during developmental stages such as adolescence and emerging adulthood, the analysis of age patterns acquires particular relevance as these stages are associated with specific developmental processes and family dynamics.

CPV comprises several manifestations, generally categorized into four types of violence. Specifically, following authors such as Cottrell (2001) and Howard and Rottem (2008), physical violence includes behaviors involving the use of force toward parents that may result in observable harm, such as hitting or pushing. Instead, financial violence refers to actions that affect or compromise parents’ economic and material resources, such as stealing money or damaging property. Psychological violence encompasses behaviors intended to cause emotional harm (e.g., denigrating, humiliating, insulting, threatening, withholding affection), whereas control/domain behaviors refer to actions aimed at exerting power over parents and limiting their autonomy (e.g., coercing, blackmailing, scaring, censoring speech, dictating topics).

Despite its growing relevance in the literature (Rogers & Ashworth, 2024), research on CPV remains limited compared to other forms of family violence. However, its increasing prevalence worldwide highlights the urgent need for greater attention from multiple sectors. Specifically, according to studies conducted in various countries, the figures in adolescents’ samples range from 3–29% for psychological CPV, 1–5% for physical CPV, 9–17% for financial CPV, and 19–65% for control/domain behaviors (Beckmann et al., 2021; Calvete & Orue, 2016; Contreras et al., 2026; Jiménez-García et al., 2022; Margolin & Baucom, 2014; Navas-Martínez et al., 2025a).

Studies on CPV have been conducted with different informants (children, parents, and professionals) although most of the accumulated knowledge comes from children’s samples and, more specifically, from children in the adolescent stage. This approach is partly due to the assumption that CPV reaches its highest frequency during mid-adolescence and tends to decrease with age (Simmons et al., 2018). This has led researchers to focus on samples up to age 18, often coinciding with legal adulthood and emancipation from the family home. However, this age-based delimitation may be insufficient in the context of current sociodemographic changes, particularly in Western societies characterized by late emancipation. In countries such as Spain, which is one of the European countries with the highest rates of delayed emancipation (Eurostat, 2024), parent–child cohabitation extends beyond age 18, extending the period in which family tensions and violent manifestations may persist. This scenario aligns with the theory of emerging adulthood (Arnett, 2000), which describes the period between ages 18 and 25 as a developmental stage differentiated from adolescence and adulthood, characterized by instability and the gradual transition toward autonomy.

Among non-emancipated young adults aged 18 to 25, greater demand for independence may coexist with different levels of economic, affective, and material dependence. This situation may give rise to specific relational dynamics that facilitate the occurrence of CPV or alter its patterns compared to those observed during adolescence. From this perspective, habitual cohabitation may be a more relevant criterion than chronological age for conceptualizing CPV (Ibabe, 2020). In fact, it has been noted that in some cases, CPV may persist or even intensify beyond adolescence (Close et al., 2024). However, research focused on the 18–25-year-old population remains scarce (Burgos-Benavides et al., 2025; Cano-Lozano et al., 2021a; Ibabe et al., 2020; Navas-Martínez et al., 2025b; Simmons et al., 2020) despite the high rates reported for this age group. Available data in these samples indicate rates of psychological CPV ranging from 44% to 67%, physical CPV from 2% to 5%, financial CPV from 15% to 37%, and control/domain behaviors from 39% to 68%.

These findings highlight the need to study CPV both specifically in young adults aged 18–25 and across a broader developmental framework encompassing adolescence and emerging adulthood. However, as this is an emerging line of research in the international literature, important gaps in knowledge still remain. One key question is whether CPV patterns change from early adolescence to late emerging adulthood. In this context, coercion theory (Patterson, 1982) stands out as a theoretical framework frequently used to explain CPV behaviors (e.g., Boxer et al., 2009; Pagani et al., 2004). This model provides an explanatory framework for the development of antisocial behavior based on family dynamics. In particular, it proposes that coercive interactions are progressively constructed through recurrent cycles of negative reinforcement between parents and children. According to this model, these coercive dynamics may begin in early stages and consolidate throughout development, giving rise to relational patterns that can be maintained or reorganized in adolescence and later stages, including emerging adulthood.

In general, age has not been a central variable in the study of CPV (Rogers & Ashworth, 2024), and the few studies that have analyzed its role as a predictor of this type of violence have done so in short and varying age ranges across studies. Age positively predicted CPV in a sample aged 13–21 (Rosado et al., 2017) and negatively in a sample aged 15–18 (Espuig et al., 2025). However, it did not prove to be a significant predictor in another sample aged 13–16 (Gao et al., 2024). These discrepancies may be due, for example, to the different age ranges studied or to possible cultural differences.

Moreover, previous studies have examined linear relationships between CPV and age, without considering potential curvilinear patterns. In the field of general delinquency, age–crime curves are well established (Farrington, 1986; Moffitt, 1993), suggesting curvilinear trends during adolescence and emerging adulthood (e.g., Fergusson & Horwood, 2002; Thulin et al., 2022; Tolan et al., 2000). This inverted U-shaped curve indicates that involvement in delinquent behavior tends to increase until mid-adolescence and then decrease. In the specific context of CPV, data from a judicial sample reinforce this general pattern. Specifically, Sheed et al. (2024) observed a decrease in the commission of CPV offenses with age, with the highest percentages in the 10–14 age group (67.34%) and the lowest in the 20–24 age group (25.56%). This suggests a decline in CPV with age in contexts of greater severity. However, in community samples, only one study (Calvete et al., 2020) analyzed the age curve of CPV. In particular, they found in a sample aged 13–17 an increase in CPV up to age 15 and a subsequent decrease. This suggests that the curvilinear pattern may also be present in CPV, insofar as changes over time can produce fluctuations in CPV levels. However, the study by Calvete et al. (2020) is limited to the adolescent period and does not clarify whether the pattern is maintained in emerging adulthood.

Another possible explanation for the inconsistent results in the literature is that in almost all studies reviewed, the relationship between CPV and age was analyzed globally rather than by specific CPV types. This issue is relevant considering, for example, that different crimes peak at different ages (Farrington, 1986). In line with this, Ibabe and Bentler (2016) found that different types of CPV are related differently to age. Specifically, age was positively related to psychological CPV and negatively related to physical CPV, although the latter was not significant. Instead, age was positively predicted CPV when analyzed globally. Similar results were obtained by Rosado et al. (2017). These findings indicate, on the one hand, that the relationship between CPV and age varies depending on the type of CPV and, on the other hand, that analyzing age in relation to CPV without differentiating its dimensions can mask relevant effects.

Likewise, the relationship between CPV and age may vary by sex. Several studies involving samples of adolescents and young adults have found differences in CPV levels between boys and girls. In general, psychological CPV and control/domain behaviors are more frequent among girls (Beckmann et al., 2021; Calvete & Orue, 2016; Contreras et al., 2026; Ibabe et al., 2020; Navas-Martínez et al., 2025a, 2025b), while financial CPV is more frequent among boys (Burgos-Benavides et al., 2025). Physical CPV is more frequent from girls toward mothers (Calvete & Orue, 2016; Jiménez-García et al., 2022; Navas-Martínez et al., 2025a) and from boys toward fathers (Navas-Martínez et al., 2025b).

Despite the above, the few studies that have explored the relationship between CPV and age have not analyzed the possible moderating role of sex in this relationship. However, in the field of juvenile violence in general, it has been suggested that the pattern of violence is similar for boys and girls across the years, although there are differences in levels of violence and in the age of onset. For example, Karriker-Jaffe et al. (2008), in a sample of adolescents aged 11–18, found that physical aggression followed curvilinear age patterns, with no differences in the shape of the curves between the sexes. That is, the shape of the curve was similar for boys and girls. However, boys showed higher initial levels than girls and remained consistently higher throughout adolescence. In contrast, no sex differences were observed in either the shape or the levels of social aggression. In addition, Shorey et al. (2017) focused on the age of onset of physical dating violence, finding an earlier onset among girls with a peak in middle adolescence, while among boys the increase occurred in late adolescence.

Overall, the literature shows that, although the pattern of CPV according to age has been examined, these analyses have been limited to short age ranges that vary across studies. As a result, it remains difficult to analyze the pattern of this type of violence across adolescence and emerging adulthood. Furthermore, the scarce available evidence does not clarify whether the association between CPV and age follows a linear or curvilinear pattern, nor whether this pattern differs among the different CPV types, or, even less so whether sex changes the shape or intensity of this relationship. As suggested in the literature review, these inconsistencies could be explained by several factors, including the use of non-comparable age ranges, cultural differences across samples, the treatment of CPV as a global construct rather than differentiating its types, and the lack of consideration of nonlinear effects and moderating variables.

In light of this, the present study aims to examine the pattern of CPV in a broad age range spanning from early adolescence to late emerging adulthood, as well as the moderating role of sex. Specifically, the following objectives were established:

1. To analyze the relationship between the CPV types (psychological, physical, financial, and control/domain behaviors) and age, evaluating both linear and quadratic effects in order to examine, for each type of CPV, whether the form of the relationship follows a linear or curvilinear pattern. H1. Based on the only study on CPV—conducted with a Spanish sample—a quadratic relationship between CPV and age is expected, with a peak around age 15 (Calvete et al., 2020). No specific hypotheses are proposed regarding the types of CPV due to the lack of studies.
2. To examine the interaction of sex in the relationship between the CPV types and age (both linear and quadratic). Although there is no evidence in the field of CPV, we expected results similar to those found in other fields of juvenile violence. Specifically: H2. Sex is expected to moderate the relationship between CPV and age (Karriker-Jaffe et al., 2008).

## 2. Materials and Methods

### 2.1. Participants

The initial sample consisted of 3270 participants. Based on the study objectives, the following selection criteria were established: being between 13 and 25 years old, having Spanish nationality, and having lived habitually with at least one parent during the past year. Participants who did not indicate their age (*n* = 2), had non-Spanish nationality (*n* = 61), and reported no contact with their father (*n* = 172) and mother (*n* = 31) during the past year were excluded from this study. Participants without parental contact were excluded because they could not provide data on the dependent variable (CPV).

The final sample comprised 3005 Spanish participants (1667 females, 1338 males) aged between 13 and 25 years. Most participants lived with both parents (89.4%) and were from the regions of Andalusia (52.5%) and Castilla-La Mancha (41.5%). The sample was divided into two subsamples: 1959 adolescents (57.8% girls) aged 13 to 17 (*M_age_* = 14.85, *SD* = 1.29) and 1046 young adults (51.1% girls) aged 18 to 25 (*M_age_* = 21.41, *SD* = 1.94). Regarding participant’s distribution by educational level, 46% (*n* = 1381) were enrolled in compulsory education and 45.7% (*n* = 1373) in post-compulsory education (non-university: 45%, *n* = 618; university: 55%, *n* = 755), while the remaining 8.3% were not enrolled in any educational program. The participants’ family characteristics are presented in Table 1, which shows that there were no statistically significant differences between adolescents and young adults in any of the categories analyzed.

### 2.2. Instruments

Ad Hoc Sociodemographic Questionnaire: It collects basic information on participant’s sex, age, nationality, school year, and family nucleus characteristics.

Child-to-Parent Violence Questionnaire, adolescent version (Contreras et al., 2019) and young adult version (Cano-Lozano et al., 2021b): Both versions assess the frequency with which a series of behaviors constituting violence toward fathers and mothers (assessed separately) have been carried out during the past year. The adolescent version includes 14 parallel items and the young adult version 19 parallel items, rated on a 5-point Likert-type response scale (0 = never; 4 = very often, has occurred six times or more).

The CPV-Q was originally developed as a 28-item instrument. In the validation studies, different item sets were retained depending on the developmental stage. Having instruments adapted to the different evolutionary stages and supported by evidence of reliability and validity ensures that the measures used are appropriate for the specific population. Accordingly, in the present study, each developmental group was assessed using its corresponding adapted version of the CPV-Q. Although both versions differ in length, they assess the same underlying theoretical dimensions.

In particular, both versions present an original factorial structure of four dimensions (physical violence, psychological violence, financial violence, and control/domain behaviors; Cano-Lozano et al., 2021b; Contreras et al., 2019). The original factor structure and the adequate psychometric properties of the CPV-Q have been replicated in adolescent samples (e.g., Jiménez-García et al., 2022; Navas-Martínez et al., 2025a; Sasaki et al., 2025; Xie et al., 2024) and young adults’ samples (e.g., Burgos-Benavides et al., 2025; Khalili et al., 2024; Navas-Martínez et al., 2025b) from several countries. The psychometric properties for the present sample are presented in the Section 3.

### 2.3. Procedure

This study presented a correlational-predictive, cross-sectional design and quantitative survey-based methodology (Montero & León, 2007), using a non-probability convenience sampling strategy to recruit all participants.

For the adolescent subsample (13–17 years, minors), ethical approval was obtained from the Ethics Committee of the University of Jaén (Spain) to conduct the study (reference: OCT.19/1.PRY), followed by authorization from public administrations and educational centers. Next, the procedure for obtaining informed consent in this subsample was as follows: Since all the adolescents were minors, in order to comply with the legal requirements in Spain, the adolescents’ parents were first informed about the study and their signed informed consent was obtained for their children’ participation. Adolescents authorized by their parents were informed about the study and their signed informed consent to participate was also obtained. The questionnaires were administered in classrooms under the supervision of trained researchers using a paper-and-pencil format.

For the young adult subsample (18–25 years, legal adults), ethical approval was obtained from the same committee (Ethics Committee of the University of Jaén) to conduct the study (reference: ABR.22/5.PRY). A snowball sampling technique was applied through the collaboration of the University of Jaén students to reach a larger number of participants, including non-university participants. The procedure for obtaining informed consent in this subsample was as follows: since all the young people were of legal age, in this case only the informed consent of the participants themselves was required. Therefore, recruited participants received the study information and signed their informed consent. Finally, they completed the questionnaires online via Google Forms.

The instructions provided to both adolescents and young adults were identical, and the questionnaires and their items were presented in the same order and format. Participation was voluntary, and the anonymity and confidentiality of the responses were guaranteed. No incentives were offered for participation.

### 2.4. Data Analysis

The significance level was set at 0.05. In a first phase, several analyses were conducted before testing the study hypotheses. Missing values were imputed using median imputation. Given the low percentage of missing data (<5%), the impact was considered minimal (Enders, 2010; Little & Rubin, 2019). With the aim of maintaining the sample size, median imputation was applied as a simple procedure.

Next, in order to ensure that the CPV-Q met the expected methodological criteria and to support the use of both versions in the subsequent age-related analyses, the psychometric properties of the instrument (father and mother scales) were examined separately for the adolescent and young adult versions. To this end, confirmatory factor analysis (CFA) of the scales was performed using the lavaan R package (Rosseel, 2012). To evaluate the goodness-of-fit of the model, the Comparative Fit Index (CFI), Tucker–Lewis Index (TLI), root mean square error of approximation (RMSEA), and standardized root mean square residual (SRMR) were used (Hair et al., 2010). Several reliability coefficients were calculated (Cronbach’s Alpha, McDonald’s Omega, Ordinal Cronbach’s Alpha, and Ordinal McDonald’s Omega), considering acceptable values ≥ 0.70 (Hair et al., 1998) or ≥0.60 (Lowenthal, 1996).

In a second phase, once it was verified that in this sample both versions of the CPV-Q maintain the same factorial structure and had similar levels of internal consistency, hypothesis testing was conducted. Mean scores were calculated of the dependent variables (CPV types: physical, psychological, financial, and control/domain behaviors) based on the corresponding items. Pearson correlations were computed between CPV types and the independent variables (age and sex). Multiple linear regressions were then performed to examine the relationship between CPV types and age, as well as the moderating role of sex in that relationship. Model assumptions were evaluated. Specifically, multicollinearity was assessed using tolerance values and variance inflation factors (VIF). The assumptions of normality and homoscedasticity were evaluated through examination of standardized residuals and residual plots.

Following standard protocol in linear regression (Cohen & Cohen, 1983) age was mean-centered. Next, quadratic terms of age were computed (Duell et al., 2018; Thulin et al., 2022), as well as interaction terms between sex and age (Aiken & West, 1991). For each CPV type, a three-step hierarchical model was estimated. In the first step, the main effects of sex and age were entered. In the second step, the quadratic term for age (age^2^) was added to examine curvilinear relationships between CPV and age. In the third step, the interaction terms (sex × age) and (sex × age^2^) were incorporated to analyze variations in the CPV-age relationship as a function of sex. Model fit was assessed using *R*^2^ and the *F* statistic. Changes in explained variance between models were examined using ΔR^2^ to determine if the additional predictors significantly improved model fit.

Finally, a sensitivity analysis was conducted to examine the robustness of the results in light of differences in item composition between the adolescent and young adult versions of the CPV-Q. Specifically, the hierarchical regression models were repeated using only the items that were identical across both versions (4 items for psychological CPV, 3 for physical CPV, 1 for financial CPV). This analysis was not performed for the control/domain behavior dimension, as the 4 items in this scale are identical in both versions. The objective was to verify whether the age-related patterns remained consistent when the analysis was restricted to strictly comparable indicators across versions.

In a third phase, to illustrate the pattern of CPV across age and possible differences between sexes, predicted values from the regressions were plotted. Specifically, for each CPV type, predictions were estimated from two equations: a linear model (sex, age, and the sex × age), and a quadratic model (sex, age, age^2^, sex × age, and sex × age^2^). All analyses described were conducted by distinguishing violence directed toward fathers and violence directed toward mothers.

## 3. Results

Confirmatory factor analyses (CFAs) of the original versions of the CPV-Q (adolescent version; Contreras et al., 2019; young adult version; Cano-Lozano et al., 2021b) showed good to excellent model fit for both the violence scale toward the father and toward the mother (see Table 2). Likewise, the reliability coefficients shown were also adequate for both the adolescent version and the young adults version, with similar values between versions. These results indicate that both versions of the CPV-Q reproduced the expected four-factor structure and showed adequate internal consistency, supporting their use for examining age-related patterns in CPV across the studied age range.

Correlational analysis (see Table 3) indicated that age was positively associated with psychological violence toward the father (*r* = 0.036, *p* < 0.05) and financial violence toward both parents (father: *r* = 0.096, *p* < 0.001; mother: *r* = 0.069, *p* < 0.001) and negatively associated with physical violence toward the mother (*r* = −0.046, *p* < 0.05) and with control/domain behaviors toward both parents (father: *r* = −0.117, *p* < 0.001; mother: *r* = −0.148, *p* < 0.001). Regarding sex, significant associations were found for some CPV types. Specifically, sex was positively associated with both psychological violence (father: *r* = 0.066, *p* < 0.001; mother: *r* = 0.090, *p* < 0.001) and control/domain behaviors toward both parents (father: *r* = 0.040, *p* < 0.05; mother: *r* = 0.039, *p* < 0.05), indicating higher levels among girls. In contrast, sex was negatively associated with financial violence toward the father (*r* = −0.049, *p* < 0.01), indicating higher levels among boys.

**Figure 1:**
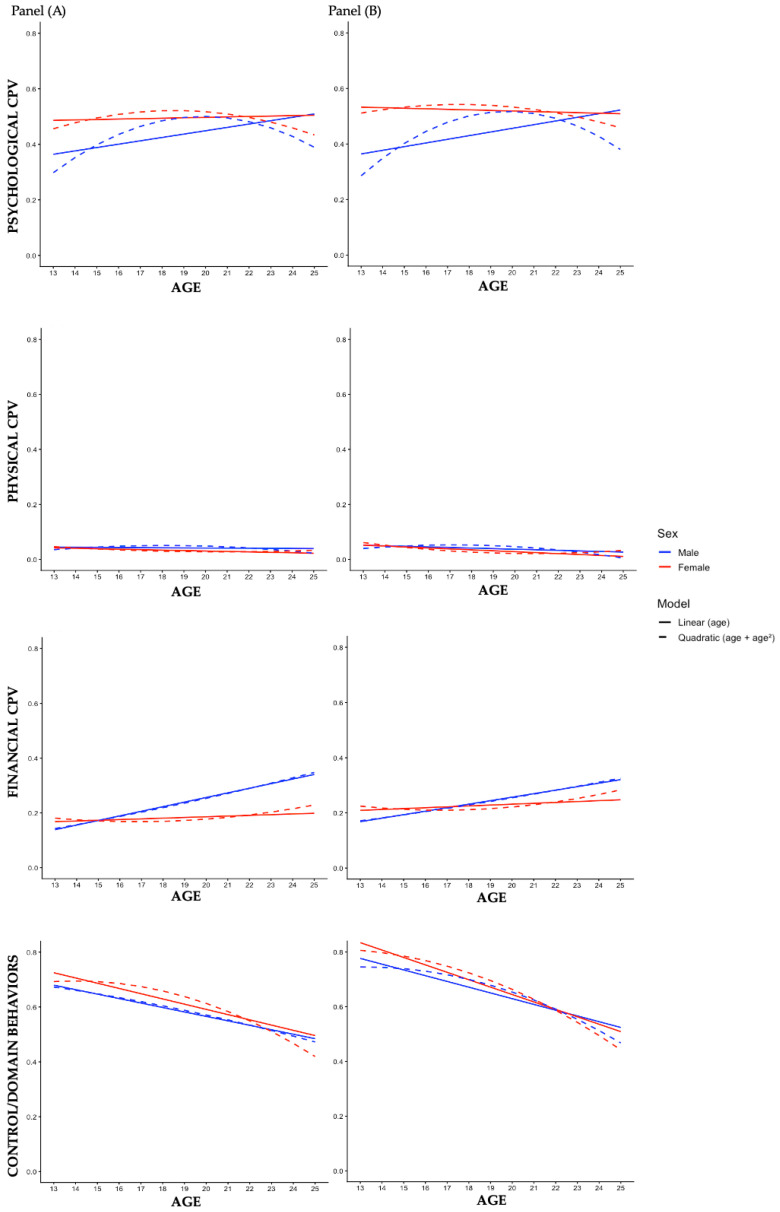
Predicted relationships of the child-to-parent violence types across age for boys and girls. Note. Panel (**A**) Child-to-father violence; Panel (**B**) Child-to-mother violence; CPV = Child-to-parent violence.

Table 4 (CPV toward the fathers’ models) and Table 5 (CPV toward the mothers’ models) present the results of the three-step hierarchical regressions analysis (Model 1: main effects of sex and age; Model 2: quadratic term of age; Model 3: interactions between sex and age) for each type of CPV, while the Figure 1 represents the predicted values. Preliminary analyses were conducted to examine whether the assumptions of the regression models were met. No evidence of problematic multicollinearity was found among the predictor variables (tolerance = 0.234–0.428; VIF = 2.335–4.266). Standardized residuals were generally centered around zero, with only a small proportion of extreme values (approximately 2% exceeding ±3), which is expected in a large sample and did not indicate substantial violations of model assumptions (Kline, 2015).

In line to the first objective of the study, in psychological CPV (see Table 4 and Table 5), Model 1 showed that age was positively and significantly related to this type of violence toward the father (*B* = 0.007, *p* < 0.05). The inclusion of the quadratic term for age (Model 2) significantly improved model fit for both the father (Δ*F*(1, 3001) = 9.197, *p* < 0.01) and mother (Δ*F*(1, 3001) = 8.996, *p* < 0.01), indicating that psychological CPV (father: *B* = −0.003, *p* < 0.01; mother: *B* = −0.003, *p* < 0.01) was curvilinearly related to age. More specifically, this type of CPV increased with age until reaching a peak (boys: 19.9 years; girls: 18.6 years) and subsequently decreased (see Figure 1). Regarding sex, girls showed higher levels of this type of violence toward fathers (*B* = 0.078, *p* < 0.001) and mothers (*B* = 0.105, *p* < 0.001) controlling for age (Model 1).

Concerning physical CPV, none of the models referring to the father (see Table 4) showed significant associations between age, sex, and this type of CPV, whereas in the models referring to the mother (see Table 5) some significant effects were observed, although they were marginal. As shown in Figure 1, levels of physical CPV toward both parents remained stable across age, suggesting an absence of both linear and quadratic relationships between this type of violence and age, and was similar between boys and girls.

Regarding financial CPV (see Table 4 and Table 5), Model 1 showed that age was positively and significantly related to this type of violence toward the father (*B* = 0.010, *p* < 0.001) and toward the mother (*B* = 0.008, *p* < 0.001), indicating an increase in financial CPV with age. The inclusion of the quadratic term of age (Model 2) did not significantly improve model fit for the father (Δ*F*(1, 3001) = 1.744, *p* = 0.188) or for the mother (Δ*F*(1, 3001) = 1.366, *p* = 0.243). Therefore, the relationship between financial CPV and age followed an upward linear trend (see Figure 1). Regarding sex, boys showed higher levels of financial violence toward fathers (*B* = −0.030, *p* < 0.05) controlling for age (Model 1).

With regard to control/domain behaviors, Model 1 showed that age was negatively and significantly related to this type of violence toward the father (see Table 4; *B* = −0.018, *p* < 0.001) and toward the mother (see Table 5; *B* = −0.024, *p* < 0.001), indicating a decrease with age. The inclusion of the quadratic term for age (Model 2) did not significantly improve model fit for the father (Δ*F*(1, 3001) = 2.149, *p* = 0.143) or the mother (Δ*F*(1, 3001) = 3.638, *p* = 0.057), and there were also no main effects of sex (Model 1). Figure 1 illustrated these effects, showing a decrease in control/domain behaviors toward fathers and mothers with age and similarly in boys and girls.

In line to the second objective of the study, the results for psychological CPV (see Table 4 and Table 5) revealed a significant interaction between sex and age (father: *B* = −0.017, *p* < 0.01; mother: *B* = −0.025, *p* < 0.001), whereas the sex × age^2^ interaction was not significant. These findings indicate that the linear age-related increase was more pronounced among boys, but the curvilinear pattern of psychological CPV across age was similar for boys and girls. As illustrated in Figure 1, the results suggest that psychological CPV tended to increase more sharply with age among boys, whereas the age-related changes were less pronounced among girls.

Regarding physical CPV, the results in Table 4 and Table 5 showed some significant effects in the interaction between sex and age. However, these effects are very small and inconsistent, in line with Models 1 and 2, which showed no relationship between physical CPV-age.

With respect to financial CPV (see Table 4 and Table 5), a significant interaction between sex and age was observed (father: *B* = −0.015, *p* < 0.01; mother: *B* = −0.011, *p* < 0.05). These results indicate that sex moderates the linear relationship between age and financial CPV, such that the increase with age is more pronounced among boys than among girls.

Concerning the control/domain behaviors, no significant interactions between sex and age were observed for CPV toward either the father or the mother (see Table 4 and Table 5). These results suggest that sex does not moderate the relationship between age and control/domain behaviors, indicating similar age-related patterns for boys and girls.

Sensitivity analyses based solely on cross-version equivalent items (see Table S1) replicated the overall pattern of the main analyses described. Specifically, the effects—both in terms of significance and direction—of psychological CPV (curvilinear relationship and a more pronounced increase in boys), physical CPV (absence of a relationship), and financial CPV (positive linear relationship and a more pronounced increase in boys) toward fathers and mothers were maintained. Only in the model of financial CPV toward the mother did age fail to reach statistical significance, although the interaction term remained significant.

Overall, the results showed distinct age-related patterns across CPV types. Psychological CPV followed a curvilinear (inverted U-shaped) pattern, financial CPV showed a positive linear increased with age, and control/domain behaviors showed a negative linear decrease with age, partially supporting Hypothesis 1. In contrast, physical CPV did not show a clear association with age. These patterns were similar for violence directed at both father and mother. Regarding sex differences, girls reported higher levels of psychological CPV toward both parents, whereas boys reported higher levels of financial CPV toward the father. Interaction effects indicated that, although general age patterns were similar for boys and girls, the slopes of psychological and financial CPV toward both parents across age were more pronounced among boys than girls, supporting Hypothesis 2.

## 4. Discussion

Age and sex are two of the least explored static risk factors in studies on CPV, and young adults’ samples remain underrepresented in the literature. The present study aimed to analyze the pattern of CPV across a broad age spanning from early adolescence to late emerging adulthood (13–25 years), as well as the moderating role of sex. Given the cross-sectional nature of the data, the results of this study are discussed in terms of patterns of association rather than developmental trajectories.

The first objective was to analyze the relationship between CPV types (psychological, physical, financial, and control/domain behaviors) and age. The results provide support for Hypothesis 1, which established significant quadratic relationships between CPV and age (Calvete et al., 2020). Specifically, age is curvilinearly related to psychological CPV (it increases until reaching a peak and then decreases). However, the form of relationship between CPV and age does not appear to follow a curvilinear pattern for all CPV types. In particular, the results show that age is positively linearly related to financial CPV (it increases with age), and negatively linearly related to control/domain behaviors (it decreases with age). These results suggest that each CPV type follows a different age pattern, whereas physical CPV is not related to age, which is consistent with previous studies (Espuig et al., 2025; Ibabe & Bentler, 2016; Rosado et al., 2017).

Regarding the practical relevance of these results, small effect sizes were observed. In context, the reduced effect size is to be expected given the use of demographic variables (age and sex) as the only predictors of a complex and multifactorial phenomenon such as CPV. Therefore, the findings have limited practical value in terms of prediction, but they are informative for describing general patterns of CPV related to age and sex differences. This contribution could aid in the formulation of future hypotheses regarding potential factors that explain changes in CPV across age.

With regard to psychological CPV, our results showed a possible pattern of progressive increase during adolescence until approximately age 19, followed by a decrease during emerging adulthood. Calvete et al. (2020) found this inverted U-shaped relationship analyzing CPV globally, and in an adolescent sample aged 13–17. The present study provides additional information of the phenomenon by suggesting that the age-related curve of CPV specifically occurs for psychologically violent behaviors, as no age curves were found for the other CPV types (physical, financial, and control/domain behaviors). Furthermore, the broad age range analyzed in this study suggests that the pattern found in Calvete et al. (2020) extends beyond adolescence. This allows for a more comprehensive understanding of how psychological CPV evolves across developmental stages. The peak age observed in this study (19 years) does not coincide with the hypothesized peak age (15 years; Calvete et al., 2020). This could be explained by the fact that the present study expands the age range analyzed and examines the shape of the relationship by separating the different CPV types.

Possible explanations for the increase in psychological CPV during adolescence may include changes in family conflict dynamics and in the parent–child relationship over time. During adolescence, parents’ attempts at behavioral and psychological control are more frequent than in later stages of development (Shah et al., 2023), which in some cases may include physical punishment or psychological aggression as disciplinary methods, factors linked to CPV at this developmental stage (e.g., Gao et al., 2024). These coercive practices by parents and violence by children may reflect dysfunctional dynamics stemming from conflicting expectations regarding authority, autonomy, and responsibilities. According to the expectancy-violation realignment model (Collins et al., 1997), these factors are an important source of conflict in adolescence. It is possible that other CPV risk factors (e.g., impulsivity, drug use, deviant peer influence) may play a more relevant role in CPV during adolescence (Rosado et al., 2017) than in emerging adulthood. Possible explanations for the decrease in psychological CPV during emerging adulthood may include the progressive development of emotional regulation and conflict resolution skills, as well as changes in the relationship with parents derived from greater autonomy and maturity in children (Arnett, 2007).

Regarding physical CPV, consistent with the findings of Ibabe and Bentler (2016) and Rosado et al. (2017), this study shows that this type of CPV is not related to age. This suggests that its development may depend more on other factors not necessarily associated with developmental changes, such as exposure to violence in the family, community, or digital contexts (e.g., Cano-Lozano et al., 2024; Navas-Martínez et al., 2025c). Exposure to family violence is one of the most relevant and consistent predictors of CPV in adolescent samples (Gallego et al., 2019), and this is likely to be so regardless of age. However, this question has not yet been explored. In this study, although levels of physical CPV are low, they remain relatively constant at all ages analyzed, suggesting that this type of violence does not disappear upon reaching age 18 but may persist in later stages. In this sense, it is necessary to analyze which factors explain not only its development but also its persistence over time.

Concerning financial CPV, our results show a pattern of progressive increase with age. Rosado et al. (2017) found in a sample aged 13–21 that age positively predicted CPV in general, but not financial CPV in particular. This result does not align with the present study, which may be explained by several reasons. For example, in Rosado et al. (2017), the sample includes ages up to 21, whereas in this study it extends to 25. In addition, recent sociodemographic and economic transformations are not comparable to those of 2017. Likewise, unlike the present study, in Rosado et al. (2017) age did not play a central role, but rather was analyzed as a control variable in a model that included variables that proved to be more relevant to CPV than age itself. Finally, the authors used a questionnaire that measured psychological and physical CPV, so financial CPV was assessed using a single item generated by the authors.

A possible explanation for the increase in financial CPV may be related to the financial needs of the children themselves, which tend to grow over time. These needs are particularly pronounced in sociocultural contexts such as Spain, where young people face great difficulties in accessing stable employment and housing. During emerging adulthood, non-emancipated individuals continue to depend economically and materially on their parents while they extend their education or face difficulties finding employment. As a result, during this developmental stage, conflicts related to money, expenses, or economic support may gain greater relevance in family dynamics (Arnett, 2000, 2007), particularly when young individuals lack their own income and parents are unable or unwilling to meet these demands.

With regard to the control/domain behaviors, the results of this study show that they decrease with age. Given that this CPV type has not been analyzed in any of the reviewed studies on CPV and age, the results cannot be compared. However, the high prevalence of these behaviors in both adolescent samples (Contreras et al., 2026; Jiménez-García et al., 2022; Navas-Martínez et al., 2025a) as in young adult samples (Burgos-Benavides et al., 2025; Cano-Lozano et al., 2021b; Navas-Martínez et al., 2025b), together with the present study’s results suggesting a different age pattern from the other CPV types. This reinforces the need to analyze control/domain behaviors as an independent CPV dimension.

Possible explanation for the decrease in control/domain behaviors may be related to changes in power relationships within the family system. The beginning of adolescence may be the period when children demand the most independence and parents impose the most restrictions on their autonomy through their position of authority (Shah et al., 2023). This period may be particularly sensitive to the appearance of behaviors aimed at defying or reverting this authority, for example, through attempts at control or dominance over parents. However, these behaviors could lose functionality as children grow due to the tendency for the parent–child relationship to evolve from more hierarchical to a more horizontal (Van-Doorn et al., 2011). This may also be due to the progressive reduction in the family’s centrality in children’s daily lives and the expansion of other life contexts outside the home (college, work, romantic partners) as they grow.

The second objective was to analyze the moderating role of sex in the relationship between CPV types and age. Consistent with Hypothesis 2 sex moderated the relationship between CPV and age, specifically the psychological and financial CPV types. As in the field of juvenile violence in general (Karriker-Jaffe et al., 2008), the results show that the linear or curvilinear relationship between CPV and age is similar for boys and girls, although violence levels differ. Specifically, girls reported higher levels of psychological CPV, whereas boys reported higher levels of financial CPV, in line with previous research (e.g., Beckmann et al., 2021; Burgos-Benavides et al., 2025; Contreras et al., 2026; Ibabe et al., 2020; Navas-Martínez et al., 2025b). Furthermore, our findings suggest that sex influences the relationship between CPV and age. Specifically, boys, comparing to girls, show a sharper increase in both psychological CPV (during adolescence)—perhaps because girls start from higher levels of this type of CPV—and financial CPV (during emerging adulthood). Likewise, the results show that the peak of psychological CPV may occur one year earlier in girls (around age 19) than in boys (around age 20). This could be explained by the fact that girls reach maturity earlier than boys, which is also consistent with Shorey et al. (2017), in which the age peak for dating violence occurred earlier in girls than in boys.

It is important to note that these interpretations are tentative, and alternative explanations should be considered, particularly in sociocultural contexts other than the Spanish one. Hence, the importance of continuing this line of research to clarify these mechanisms.

### Limitations, Contributions, and Future Research

The literature highlights the need of using instruments adapted to different developmental stages. In the present study, each developmental stage was assessed using a specifically adapted version of the CPV-Q. Both versions assess the same theoretical dimensions through different numbers of items. This difference in the number of items between the two versions, while attributable to evolutionary differences, nevertheless represents a significant methodological limitation of the present study. In the present sample both versions of the CPV-Q showed adequate factorial fit and reliability, and presented similar values between versions. However, the results should be interpreted with caution, as the use of different versions of the instrument across age groups may have introduced some measurement-related differences. Therefore, the observed age-related patterns cannot be attributed exclusively to developmental changes without considering the potential influence of measurement characteristics specific to each version. This limitation has been mitigated through a sensitivity analysis that replicated the patterns observed when the CPV was operationalized using measures that are strictly comparable across both versions. However, financial CPV toward the mother was not associated with age in this analysis, unlike the results of the main analysis. Overall, the observed patterns require replication in future studies.

Another aspect to consider when interpreting the results is the relatively small proportion of variance explained by the regression models. Although several effects reached statistical significance, the *R*^2^ values were modest across models. This is not unexpected given the multicausal nature of CPV, which is influenced by a wide range of individual, family, and social factors (Cano-Lozano et al., 2024; Del Hoyo-Bilbao et al., 2020). Given the aims of this study, only age and sex were included as predictors. Consequently, the results should be interpreted in the context of the objectives of this study.

On the other hand, the results are based on cross-sectional data. In this regard, although cross-sectional analyses allow the identification of associations between age and certain behaviors, they do not allow for the inference of developmental trajectories at the individual level or for establishing causal or within-person developmental changes; therefore, the aims of this study as well as the explanations offered in the discussion should be tested in longitudinal samples.

Regarding the participants, given the non-probability sampling, the generalizability of the results to the reference population is limited, so future research should replicate the study using probability sampling designs. In this regard, participants come mainly from two regions of Spain, so the results are not fully generalizable to the rest of the Spanish population, and should not be extrapolated to different sociocultural contexts, especially those in which prolonged cohabitation and the delayed emancipation of children do not extend beyond the age of 18. It would be important to replicate the study design in other Spanish regions and in other countries to explore potential transcultural differences in the age pattern of CPV. Finally, the data rely exclusively on self-reports from children, which may be subject to biases such as social desirability or underreporting, particularly in the case of sensitive behaviors like violence. This was addressed by making the responses anonymous. However, it would be advisable for future studies to complement this information with reports from other informants, such as parents.

With respect to procedure, adolescents completed the questionnaires in paper-and-pencil and under supervision, whereas young adults completed them online and without direct supervision. These procedural differences could constitute a potential source of bias, so the results should be interpreted with caution. However, numerous studies have shown that the quality of data of digital surveys is comparable to that of traditional paper surveys (e.g., metanalysis by Gwaltney et al., 2008), and that digital surveys reduce item nonresponse compared to paper-based surveys (Hallfors et al., 2000). This was addressed in the adolescent subsample through in-person supervision by trained researchers, since supervision is recommended collecting data from adolescents and in large groups (Stefes, 2024). In young adults the unsupervised digital survey was chosen, given that no differences have been found in digital modes compared to in-person modes in these samples (Mühlböck et al., 2017). Nevertheless, future research should aim to use more homogeneous data collection procedures to fully rule out potential methodological biases.

Despite these limitations, this study is one of the few that analyzes age and sex in relation to CPV. More importantly, to our knowledge, it is the only one to analyze the pattern of each CPV type from early adolescence through late young adulthood across a broad age range. The study provides valuable information that improves understanding of this type of family violence. In particular, it is suggested, first, that CPV is not limited to adolescence, but also occurs in non-emancipated young adults; second, that each type of CPV seems to follow a different age-related pattern; and third, that sex appears to influence the relationship between some types of CPV and age.

These findings may have some practical implications. From an academic perspective, the results underscore the need both to broaden the developmental period from which CPV is analyzed and to differentiate CPV types when studying it. Nonreflecting the complexity of the phenomenon in research impedes advances in knowledge of this serious social problem. Likewise, this study may serve as a guide for future research on which CPV risk and protective factors should be explored at each developmental stage, especially in emerging adulthood where evidence remains scarce.

From an applied perspective, although further research is needed, the results could be useful for guiding the early detection of CPV cases and for allocating preventive resources. In this sense, understanding age-related patterns of CPV could facilitate the identification of warning signs, such as the earlier emergence of psychological CPV behaviors at younger ages and financial CPV behaviors at older ages. In terms of intervention, these patterns could suggest the advisability of adapting preventive strategies to the developmental stage. For example, by prioritizing different types of violence according to age and adjusting preventive content to the most likely manifestations in each period. Likewise, the results related to sex indicate that, in some types of CPV, there may be differences in levels and evolution with age. This would support the usefulness of incorporating a gender perspective in the design of interventions, considering possible differences in the expression of these behaviors between boys and girls. However, since the results of this study are based on a Spanish sample, these implications should be interpreted with caution in diverse cultural contexts, where the age of emancipation and CPV age patterns may differ.

Rather than aiming to produce robust predictive models, this study was designed to examine the pattern of CPV across age. The findings suggest the pertinence of considering age as a relevant variable when studying CPV as a control variable or as a factor to be explicitly modeled. First, it is important to identify which are the factors that explain the changes in the CPV pattern across adolescence and emerging adulthood. Second, it is necessary to understand how risk and protective factors may operate across developmental stages in the development of CPV. Accordingly, future studies on risk and protective factors for CPV should examine whether their effects are independent of age or, on the contrary, influenced by it, while addressing the role of sex. Finally, further efforts are needed to design CPV prevention strategies that take into account children’s developmental stage and incorporate a gender perspective.

## 5. Conclusions

The present findings contribute to expanding current knowledge on child-to-parent violence (CPV), although they should be interpreted with caution until further research becomes available. In particular, this study suggests that CPV is not limited to adolescence, but is also present among non-emancipated young adults. Furthermore, the results suggest that the different CPV types present differentiated age-related patterns and that sex influences some of these associations. These findings underscore the importance of analyzing CPV by considering both the developmental stage and the specific type of CPV and the role of sex, as well as expanding the age range typically studied in the literature. They also highlight the need for future research to focus on the analysis of stage-specific CPV risk and protective factors, particularly in emerging adulthood, where empirical evidence remains limited. From an applied perspective, the results may contribute to improving early detection of CPV, anticipating potential changes in its patterns, and informing guide prevention strategies tailored to the most prevalent forms of CPV at each developmental stage.

## Figures and Tables

**Table 1:** Differences between adolescents and young adults in sociodemographic variables.

	Adolescents *N* = 1959 % (*n*)	Young Adults *N* = 1046 % (*n*)	χ^2^	φ/Cramer’ V
Family type			<0.001	<0.001
Biological	99.4 (1923)	99.3 (1039)		
Adoptive	0.6 (12)	0.7 (7)		
Parent’s marital status			9.109	0.055
Married	83.6 (1633)	83.8 (877)		
Live together	3.0 (59)	1.3 (14)		
Divorced/separated	13.1 (255)	14.4 (151)		
Widower	0.3 (5)	0.3 (3)		
Never live together	0.1 (1)	0.1 (1)		
Number of siblings			1.630	0.023
One	12.7 (249)	13.3 (139)		
Two	63.9 (1251)	63.0 (659)		
Three	17.5 (343)	18.6 (195)		
Four or more	5.9 (116)	5.1 (53)		
Economic levels (monthly)			6.984	0.053
Less than 1000 euros	6.1 (86)	8.8 (92)		
Between 1000 and 2000 euros	48.7 (687)	48.7 (509)		
Between 2000 and 3000 euros	31.4 (443)	29.7 (311)		
More than 3000 euros	13.8 (194)	12.8 (134)		

**Table 2:** Fit indexes and reliability of the scales used in the study.

CPV-Q Version	Scales	Χ^2^	*df*	*p*	CFI	TLI	SRMR	RMSEA	α	α_o_	ω	ω_o_
Adolescents	CPV-F	222.260	71	<0.001	0.969	0.960	0.055	0.033	0.680	0.846	0.770	0.884
	CPV-M	336.144	71	<0.001	0.949	0.935	0.057	0.044	0.713	0.866	0.798	0.898
Young Adults	CPV-F	545.88	146	<0.001	0.960	0.953	0.082	0.051	0.841	0.948	0.907	0.963
	CPV-M	461.407	146	<0.001	0.963	0.957	0.086	0.045	0.833	0.946	0.904	0.963

*Note.* CPV-Q = Child-to-Parent Violence Questionnaire; CPV-F = child-to-father violence; CPV-M = child-to-mother violence; *df* = degree of freedom; CFI = comparative fit index, TLI = Tucker–Lewis index, RMSEA = root mean square error of approximation, SRMR = standardized root mean square residual.

**Table 3:** Correlations between the dependent and independent variables of the study.

	1	2	3	4	5	6	7	8	9	10
1. CPV-F Psychological	-									
2. CPV-F Physical	0.318 ***	-								
3. CPV-F Financial	0.357 ***	0.281 ***	-							
4. CPV-F Control/Domain	0.348 ***	0.204 ***	0.284 ***	-						
5. CPV-M Psychological	0.764 ***	0.227 ***	0.355 ***	0.304 ***	-					
6. CPV-M Physical	0.274 ***	0.786 ***	0.262 ***	0.198 ***	0.332 ***	-				
7. CPV-M Financial	0.318 ***	0.236 ***	0.820 ***	0.267 ***	0.397 ***	0.268 ***	-			
8. CPV-M Control/Domain	0.306 ***	0.178 ***	0.290 ***	0.837 ***	0.357 ***	0.219 ***	0.339 ***	-		
9. Sex	0.066 ***	−0.017	−0.049 **	0.040 *	0.090 ***	−0.009	−0.003	0.039 *	-	
10. Age	0.036 *	−0.016	0.096 ***	−0.117 ***	0.026	−0.046 *	0.069 ***	−0.148 ***	−0.076 ***	-

*Note.* CPV-F = child-to-father violence; CPV-M = child-to-mother violence. Sex: 1 = females. * *p* < 0.05, ** *p* < 0.01, *** *p* < 0.001.

**Table 4:** Hierarchical regression models examining the effects of age and sex on the child-to-father violence types.

		Model 1			Model 2			Model 3		
CPV Types	Predictors	*B*	*t*	95% CI	*B*	*t*	95% CI	*B*	*t*	95% CI
Psychological	Sex	0.078 ***	3.767	0.037, 0.119	0.075 ***	3.640	0.035, 0.116	0.050	1.580	−0.012, 0.112
	Age	0.007 *	2.272	0.001, 0.013	0.014 ***	3.666	0.006, 0.021	0.024 ***	4.137	0.012, 0.035
	Age^2^				−0.003 **	−3.031	−0.005, −0.001	−0.004 **	−2.995	−0.007, −0.001
	Sex × Age							−0.017 **	−2.297	−0.032, −0.003
	Sex × Age^2^							0.002	1.089	−0.002, 0.006
	*R* ^2^	0.006			0.009			0.011		
	*F*	9.076 ***			9.129 ***			6.579 ***		
Physical	Sex	−0.007	−0.984	−0.022, 0.008	0.008	−0.994	−0.022, 0.007	−0.018	−1.604	−0.041, 0.004
	Age	−0.001	−0.967	−0.003, 0.001	−0.001	−0.594	−0.004, 0.002	0.001	0.553	−0.003, 0.005
	Age^2^				<0.001	−0.262	−0.001, 0.001	−0.001	−1.134	−0.002, <0.001
	Sex × Age							−0.003	−1.242	−0.009, 0.002
	Sex × Age^2^							0.001	1.264	<−0.001, 0.002
	*R* ^2^	<0.001			<0.001			0.001		
	*F*	0.885			0.612			0.754		
Financial	Sex	−0.030 *	−2.304	−0.056, −0.004	−0.029 *	−2.246	−0.055, −0.004	−0.037	−1.858	−0.076, 0.002
	Age	0.010 ***	5.114	0.006, 0.013	0.008 **	3.187	0.003, 0.012	0.016 ***	4.487	0.009, 0.023
	Age^2^				0.001	1.318	<−0.001, 0.002	<0.001	0.291	−0.001, 0.002
	Sex × Age							−0.015 **	−3.232	−0.025, −0.006
	Sex × Age^2^							0.001	0.522	−0.002, 0.003
	*R* ^2^	0.011			0.012			0.017		
	*F*	16.730 ***			11.730 ***			9.895 ***		
Control/ Domain	Sex	0.034	1.707	−0.005, 0.072	0.032	1.644	−0.006, 0.071	0.055	1.834	−0.004, 0.113
	Age	−0.018 ***	−6.309	−0.023, −0.012	−0.014 ***	−4.033	−0.021, −0.007	−0.015 **	−2.791	−0.026, −0.004
	Age^2^				−0.001	−1.464	−0.003, <0.001	<−0.001	−0.316	−0.003, 0.002
	Sex × Age							0.001	0.124	−0.013, 0.015
	Sex × Age^2^							−0.002	−0.998	−0.006, 0.002
	*R* ^2^	0.015			0.015			0.016		
	*F*	22.310 ***			15.590 ***			9.635 ***		

*Note.* CPV = child-to-parent violence. Sex: 1 = females. * *p* < 0.05, ** *p* < 0.01, *** *p* < 0.001.

**Table 5:** Hierarchical regression models examining the effects of age and sex on the child-to-mother violence types.

		Model 1			Model 2			Model 3		
CPV Types	Predictors	*B*	*t*	95% CI	*B*	*t*	95% CI	*B*	*t*	95% CI
Psychological	Sex	0.105 ***	5.080	0.065, 0.146	0.103 ***	4.954	0.062, 0.143	0.060	1.912	−0.002, 0.012
	Age	0.005	1.838	<−0.001, 0.011	0.012 ***	3.303	0.005, 0.020	0.027 ***	4.693	0.016, 0.038
	Age^2^				−0.003 **	−2.995	−0.005, −0.001	−0.005 ***	−3.541	−0.008, −0.002
	Sex × Age							−0.025 ***	−3.354	−0.040, −0.011
	Sex × Age^2^							0.004	1.804	<−0.001, 0.007
	*R* ^2^	0.009			0.012			0.016		
	*F*	13.960 ***			12.320 ***			9.694 ***		
Physical	Sex	−0.005	−0.711	−0.020, 0.009	−0.005	−0.710	−0.020, <0.001	−0.023 *	−1.970	−0.045, <−0.001
	Age	−0.003 *	−2.572	−0.005, −0.001	−0.003 *	−2.013	−0.005, <−0.001	<−0.001	−0.001	−0.004, 0.004
	Age^2^				<−0.001	−0.002	−0.001, 0.001	−0.001	−1.492	−0.002, <0.001
	Sex × Age							−0.005	−1.739	−0.010, <0.001
	Sex × Age^2^							0.001 *	1.998	<0.001, 0.003
	*R* ^2^	0.002			0.002			0.004		
	*F*	3.441 *			2.293			2.254 *		
Financial	Sex	0.002	0.105	−0.027, 0.030	0.002	0.155	−0.026, 0.031	−0.008	−0.371	−0.051, 0.035
	Age	0.008 ***	3.795	0.004, 0.012	0.006 *	2.247	0.001, 0.011	0.012 **	3.054	0.004, 0.020
	Age^2^				0.001	1.168	−0.001, 0.002	<0.001	0.198	−0.002, 0.002
	Sex × Age							−0.011 *	−2.119	−0.022, −0.001
	Sex × Age^2^							0.001	0.639	−0.002, 0.004
	*R* ^2^	0.005			0.005			0.007		
	*F*	7.219 ***			5.268 *			4.217 ***		
Control/ Domain	Sex	0.032	1.535	−0.010, 0.074	0.031	1.453	−0.011, 0.071	0.030	0.950	−0.032, 0.093
	Age	−0.024 ***	−8.034	−0.030, −0.018	−0.020 ***	−5.110	−0.027, −0.012	−0.016 **	−2.698	−0.027, −0.004
	Age^2^				−0.002	−1.908	−0.004, <0.001	−0.002	−1.388	−0.005, 0.001
	Sex × Age							−0.007	−0.936	−0.022, 0.008
	Sex × Age^2^							<0.001	0.012	−0.004, 0.004
	*R* ^2^	0.023			0.024			0.024		
	*F*	34.590 ***			24.300 ***			14.860 ***		

*Note.* CPV = child-to-parent violence. Sex: 1 = females. * *p* < 0.05, ** *p* < 0.01, *** *p* < 0.001.

## Data Availability

The raw data supporting the conclusions of this article will be made available by the authors, without undue reservation.

## Supplementary Materials

The following supporting information can be downloaded at: https://www.mdpi.com/article/10.3390/ejihpe16050070/s1, Table S1: Sensitivity analysis based on common-item indicators of the adolescents and young adults versions of the Child-to-Parent Violence Questionnaire.
