# Supplementary material for: Age-Related Patterns in Child-to-Parent Violence Across Adolescence and Emerging Adulthood
Source: Eur J Investig Health Psychol Educ. 2026 May 17;16(5):70. doi: 10.3390/ejihpe16050070 (PMC13206349; doi:10.3390/ejihpe16050070)
Supplement: Supplementary file 1 [file ejihpe-16-00070-s001.zip › ejihpe-4233214-supplementary.pdf]

Table S1. Sensitivity analysis based on common-item indicators of the adolescents and young adults versions of the Child-to-Parent Violence Questionnaire

		Child-to-Father Violence			Child-to-Mother Violence		
		Model 3			Model 3		
CPV Types	Predictors	*B*	*t*	95% CI	*B*	*t*	95% CI
Psychological	Sex	.056	1.777	-.006, .118	.408	1.920	-.001, .121
	Age	.004	0.731	-.007, .015	.003	0.551	-.008, .014
	Age^2	-.003*	-2.257	-.006, -.001	-.004**	-2.991	-.007, -.001
	Sex × Age	-.019*	-2.533	-.034, -.004	-.029***	-3.814	-.043, -.014
	Sex × Age^2	.001	0.441	-.003. .005	.003	1.308	-.001, .006
	R^2	.015			.029		
	F	9.231***			18.103***		
Physical	Sex	-.020	-1.693	-.044, .003	-.026*	-2.165	-.049, -.002
	Age	.001	-0.627	-.003, .006	.000	0.081	-.004, .004
	Age^2	-.001	-1.266	-.002, .000	-.001	-1.790	-.002, .000
	Sex × Age	-.004	-1.372	-.010, .002	-.005	-1.872	-.011, .000
	Sex × Age^2	.001	1.217	-.001, .002	.002*	2.124	.000, .003
	R^2	.002			.005		
	F	.978			2.714*		
Financial	Sex	-.060	-1.867	-.124, .003	-.026	-0.718	-.097, .045
	Age	.013*	2.191	.001, .024	.012	1.892	.000, .025
	Age^2	.000	-0.125	-.003, .003	-.001	-0.339	-.004, .003
	Sex × Age	-.032***	-4.147	-.048, -.017	-.033***	-3.799	-.005, -.016
	Sex × Age^2	.003	1.672	-.001, .007	.003	1.173	-.002, .007
	R^2	.008			.006		
	F	4.697***			3.778**		

Note. CPV = child-to-parent violence. Sex: 1 = females. Models were re-estimated using only items worded identically in both adolescents and young adults versions of the CPV-Q (psychological = 4 items; physical = 3 items; financial = 1 item). Control/domain behaviors were not re-estimated because both versions are identical items. * $p < .05$, ** $p < .01$, *** $p < .001$.
